# Speech perception in the Specific Learning Disorder with and without Persistent Speech Sound Disorder

**DOI:** 10.1590/2317-1782/20242024034en

**Published:** 2024-10-04

**Authors:** Mariana Martins Appezzato, Clara Regina Brandão de Avila

**Affiliations:** 1 Programa de Pós-graduação em Distúrbios da Comunicação Humana, Escola Paulista de Medicina – EPM, Universidade Federal de São Paulo – UNIFESP - São Paulo (SP), Brasil.; 2 Departamento de Fonoaudiologia, Escola Paulista de Medicina – EPM, Universidade Federal de São Paulo – UNIFESP - São Paulo (SP), Brasil.

**Keywords:** Speech Perception, Speech Disorders, Specific Learning Disorder, Education, Primary and Secondary, ROC Curve

## Abstract

**Purpose:**

Investigate if speech perception skills can differentiate school children with Specific Reading Disorders (SRD) with and without Persistent Speech Sound Disorders (PSSD).

**Methods:**

80 children, regularly enrolled in the 2nd (N=1), 3rd (N=28), 4th (N=29), 5th (N=15) and 6th (N=7) grades participated in the study. Control Group (CG) (N=48): no complaints, no speech alteration; and Resarch Group (RG) (N=32) – with SRD, RGI (N=15) without PSSD and RGII (N=17) with PSSD. Two tests evaluated auditory input reception: Simplified evaluation of auditory processing; and Perception task of nonwords, with Portuguese language structure (DNPLS). Data was analyzed by: Likelihood Ratio Test, Kruskal-Wallis test, Dunn test with Bonferroni correction, Mann-Whitney test, Spearman correlation, and construction of a ROC curve to obtain a threshold value for the correct answers in the perception of non-words test.

**Results:**

Control and RGI showed higher correct answer scores than RGII. There was no difference between the correct answer distributions of the Control and RGI, and RGI and RGII in the test of DNPLS and the number of correct answers in the CG was higher than in the RGII.

**Conclusion:**

The ability to discriminate non-words enabled the differentiated between school-aged children with SRD associated with PSSD and typical children, thus characterizing this group for presenting a number of correct answers lower than 30.5, considering the task proposed to discriminate non-words. These results suggest that the presence of PSSD worsens the performance in speech perception of the schoolchildren with SRD.

## INTRODUCTION

The ability to perceive a single phoneme in connected speech is a metalinguistic competence demanded in learning the alphabetic principle^(1,2)^. Production and speech perception changes can interfere with the acquisition of phonological contrasts and the categorical organization of mental representations of linguistic segments, hindering the development of phonological awareness^(3-5)^. Scientific evidence indicates that it is necessary to consider underlying issues to this ability to mentally manipulate the sounds of the speech chain^(6)^, especially when evaluating a child with complaints and signs of some Specific Learning Disorder (SLD)^(7)^. In such cases, more than thinking about the deficit of the metacognitive capacity to manipulate phonemes and connect them to letters, we should consider the conditions and characteristics of the phonological representations from speech development^(8)^ and how it is organized and perceived.

Thus, it is not uncommon to find children with reading or writing disorders who manifest a speech sound disorder in a co-occurrence or association^(9-12)^. The current basis of evidence suggests that the presence of speech sound changes increases the propensity to undertake more significant efforts to acquire literacy skills^(13-15)^. Speech sound disorder at the time of literacy can indicate its persistence, and interfere with or restrict communication, negatively impacting social interaction or academic success^(7,9)^.

Specific Learning Disorders may display a spectrum of cognitive-linguistic skill impairments manifesting in different language domains^(7)^. Not infrequently, they are observed from more severe speech sound disorders^(14)^ to subclinical impairments involving specific errors^(15)^ or phonological processes not yet solved^(9,16)^ following the SLD.

Since they are still observed at school age (late, after the finalization of speech acquisitions) and, considering the characteristics of their production, these speech changes are considered Persistent Speech Sound Disorders (PSSD)^(15)^. They are the outcome of a development that deviated from the expected, in time and order of acquisition, or even with distortions^(15,16)^. Researchers^(17)^ agree that the risk of showing SLD increases as more language skills and speech are altered at ages that precede or focus on entry into elementary school. SLD may be often associated with auditory processing and speech perception impairments^(18)^.

The common denominator of specific learning disorders, with loss in reading and writing^(7)^, is a phonological information processing deficit, which manifests in various skills and tasks to recognize and write words. The underlying mechanisms that drive this deficit are not yet fully known^(6)^. Speech perception studies in these cases are the least frequent. So, it seems relevant to know which mechanism involved in sensory or auditory perceptual inputs is more fragile in speech perception^(19)^, especially when changes in the speech system are identified beyond the expected age.

Changes related to speech perception difficulties can attach harm to sound discrimination, that is, attention to the acoustic differences of speech sounds; categorical perception or classification of speech sounds to judgment categories; error detection; or the ability to perceive whether or not a speech sound belongs to own language; or producing words based on the native language^(19-21)^.

Speech perception deficits are commonly reported in the SLD, and overlapping speech-to-reading changes deteriorate the conditions of auditory inputs, especially in perceiving the acoustic characteristics of the phonemes^(6)^ and proceeding to any corrections. However, the longitudinal evidence that poor speech perception compromises reading learning is still scarce.

As a result, this study focuses on investigating specific characteristics of speech perception of Elementary School I students diagnosed with SLD. At data collection, some students evidenced PSSD in association with the primary condition and formed an investigation subgroup. This research was conducted under a psycholinguistic model^(21)^ and evaluated the capacity for categorical perception of speech sounds by applying a list of pseudowords for discrimination and phonemic classification^(22)^.

We examined speech perception in different phonological deficit profiles against normal development. Thus, questioning whether PSSD can deteriorate the speech perception of students with SLD has defined this research. From this issue and the evidence of the literature, then, we raised the hypothesis that, in SLD, the ability to categorically perceive speech sounds is deficient compared to typical development students, and it will be worse when in association with the PSSD.

## METHODS

This cross-sectional, quantitative analysis study was submitted to (CAAE: 47313115.5.0000.5505) and approved by CEP-UNIFESP (N° 1415919). All participants signed the Informed Consent Form (ICF), the Child Assent Term (CAT), and the School Consent (SC).

### Participants

This purposeful sample of students comprised 80 schoolchildren (from 08 years and 2 months to 11 years and 8 months / Mean=9.3 years; SD=0.9) from São Paulo’s Public School Network. We established the regular enrollment in the 2^nd^ to 6^th^ grades of Elementary School I from public schools as a general inclusion criterion. We excluded complaints or indicators of sensory (hearing or visual) deficits, neurological disorders, cognitive deficits mentioned by teachers or their parents, craniomaxillofacial anomalies or alterations, absence of cochleo-palpebral reflex in the simplified evaluation of auditory processing^(23)^, and not signing ICF or CAT as exclusion criteria in the sample.

The 80 schoolchildren were grouped as follows: Control Group (CG) (n=48 students with typical learning development) and Research Group (RG) (n=32 students with specific learning disorders). The RG has been regrouped in: RGI (n=15 students with SLD) and RGII (n=17 students with SLD and PSSD). This group’s performance was compared to RGI (SLD) and CG to study the characteristics of auditory-linguistic perception in the presence of PSSD.

The RG participants were under care at the Evaluation and Therapy Outpatient Clinic of Reading and Writing Disorders and received a multidisciplinary diagnosis of SLD at the time of data collection. The RGII overlapped in the co-occurrence of the speech therapy diagnosis of PSSD.

### Procedures

The following tests and exams were applied in the evaluation of all participants:

- Assessment of Speech Sounds ABFW - Phonology^(24)^ - naming and imitation tests were applied to survey the phonetic inventory, investigate phonological processes and calculate the Percentage of Correct Consonants index^(25)^. The evaluation allowed us to identify, classify, and separate, among the children in the RG, those with alterations in speech sounds in conjunction with SLD. (Descriptive data in supplementary material – Charts S1 and S2);
- The Simplified Auditory Processing Assessment (ASPAC)^(23)^ assessed the first level of auditory-perceptual input after the sensory input^(7)^ since it also evaluates the perception of linguistic sounds. Investigating the cochleo-palpebral reflex allowed us to exclude children at risk of showing auditory problems that could affect the results of applying the following tests;
- Pseudoword Perception Task with Portuguese Language Structure (TDP)^(22)^: allows us to evaluate the ability to discriminate speech sounds (without reference to lexical representation) and to perceive speech sounds^(4,20)^ categorically. Eighteen test items composed this task^(22)^.

These two procedures for assessing auditory-linguistic perception were applied and analyzed under the procedures and parameters described by the authors^(22,23)^.

Data were collected individually in a single assessment session lasting 50 minutes. The CG children were assessed in the school’s resource room, provided by the school’s administration. The RG was assessed in a quiet room in the Outpatient Clinic.

The Kruskal-Wallis test (p<0.05) and the Chi-square test (p<0.05) analyzed, respectively, the distributions of age and probability of sex in the three groups. The measures of central tendency and dispersion of hits in the Pseudoword Perception Task with Portuguese Language Structure (TDP) per the group and the comparison of the groups were studied through ANOVA with one independent factor or Kruskal-Wallis test.

We investigated the sensitivity and specificity for identifying individuals with SLD and PSSD. To characterize the groups, we established the cutoff point with the most significant balance between these two parameters. We used the total number of hits in the TDP to identify individuals in RGII to calculate the ROC curve. The CG and RGI were gathered in a single group because they did not show any difference in this test. The statistical significance level adopted in the data analysis was 5% (p≤0.05). We used the SPSS Statistics software, version 25.0 (IBM Corp., Armonk, NY, USA). The bias-corrected and accelerated method based on 2,000 Bootstrap samples was used to calculate the 95% confidence intervals. The analysis was performed using SPSS – version 18, Minitab – version 18, and R 3.5.1.

## RESULTS

The three groups were similar (p=0.700) regarding age distribution. The children in the CG were distributed from the 3^rd^ to the 5^th^ grade; those in the RGI, from the 3^rd^ to the 6^th^ grade; and those in RGII, from the 2^nd^ to the 6^th^ grade. On the other hand, the distributions of the percentages of children by school year in the three groups differed (per the supplementary material – Tables S1, S2 and S3).

Comparing the performance in auditory perception in the Sound Localization, Non-Linguistic Sound Sequence Memory, and Linguistic Sound Sequence Memory tasks (Table 1) showed no difference between the Research Groups. For subsequent analyses, both research groups were considered a single group compared to the CG. The latter performed better in these three auditory perception tasks.

**Table 1:** Frequency distributions and percentages of total hits in the Sound Localization, Non-Linguistic Sound Sequence Memory, and Linguistic Sound Sequence Memory Tests in the RGI, RGII, and CG

Tests		RGI	RGII	CG	Total
	Number of hits				
SL	0	2	0	0	2
		13.30%	0.00%	0.00%	2.50%
	1	13	17	0	30
		86.70%	100%	0.00%	37.50%
	3	0	0	3	3
		0.00%	0.00%	6.30%	3.80%
	4	0	0	13	13
		0.00%	0.00%	27.10%	16.30%
	5	0	0	32	32
		0.00%	0.00%	66.70%	40.00%
NLSSMT	0	1	2	0	3
		6.70%	11.80%	0.00%	3.80%
	1	14	15	3	32
		93.30%	88.20%	6.30%	40.00%
	2	0	0	15	15
		0.00%	0.00%	31.30%	18.80%
	3	0	0	30	30
		0.00%	0.00%	62.50%	37.50%
LSSMT	0	2	2	0	4
		13.30%	11.80%	0.00%	5.00%
	1	13	15	0	28
		86.70%	88.20%	0.00%	35.00%
	2	0	0	13	13
		0.00%	0.00%	27.10%	16.30%
	3	0	0	35	35
		0.00%	0.00%	72.90%	43.80%
	Total	15	17	48	80
		100.00%	100%	100%	100.00%

Likelihood Ratio Test (p=0.424): RG1=RG2 | Chi-square test: CG>RG (p<0.001)

**Caption:** SL = Sound Localization; NLSSMT = Non-Linguistic Sound Sequence Memory Test; LSSMT = Linguistic Sound Sequence Memory Test

Thes CG and RG groups showed different performances in the TDP (Table 2). The calculation of the effect size of the difference between the groups using the r coefficient indicated that this difference was found between the CG and RGII groups (p=0.035, r=0.313).

**Table 2:** Descriptive values ​​and comparative analysis of the groups regarding the number of hits in the TDP

Test	Group	n	Mean	SD	Median	Min.	Max.	p
TDP	CG	48	31.00 [29.92. 31.94]	3.48	31.50 [31.00. 32.50]	16.00	34.00	**0.039** *
	RGI	15	30.47 [27.80. 32.27]	4.47	31.00 [31.00. 33.00]	16.00	34.00	
	RGII	17	29.35 [28.12. 30.59]	2.76	30.00 [29.00. 30.00]	24.00	33.00	

Kruskal-Wallis test (^b^)

* Statistically significant value at the 5% level (p ≤ 0.05)

**Caption**: SD: Standard Deviation; Min.: Minimum; Max.: Maximum

Since the CG and RGI groups did not differ in the TDP test, they were grouped as a single group. The ROC curve characterized the ability of the total number of hits in TDP to identify individuals from the RGII.

**Figure 1:**
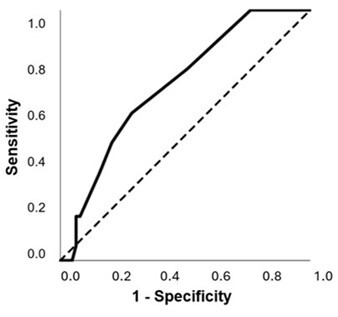
ROC curve for the total number of hits in the TDP. Note: The dashed line indicates a hypothetical test with no discriminating ability. Area Under the Curve (AUC) = 0.698 (95% CI: [0.570, 0.827]).

An area under the curve (AUC) value of 0.698 indicated that an individual from RG II (with SLD and PSSD) has a 69.8% probability of having a lower number of hits in the TDP, when compared to a student from RGI or CG, that is, without Persistent Speech Sound Disorder (Figure 1).

The cutoff point with the most significant balance between sensitivity and specificity for the number of hits in the TDP was 30.50, in which the sensitivity was 58.82% and the specificity was 71.43% (Table 3).

**Table 3:** Cutoff points for the number of hits in the TDP and associated sensitivity, specificity, and efficiency values

Cutoff point	Sensitivity (%)	Specificity (%)	False-positive (%)	False-negative (%)	Efficiency (%)	J
18.50	0.00	96.83	3.17	100.00	48.41	-0.032
22.50	0.00	95.24	4.76	100.00	47.62	-0.048
24.50	5.88	93.65	6.35	94.12	49.77	-0.005
26.00	17.65	93.65	6.35	82.35	55.65	0.113
27.50	17.65	92.06	7.94	82.35	54.86	0.097
28.50	35.29	84.13	15.87	64.71	59.71	0.194
29.50	47.06	79.37	20.63	52.94	63.21	0.264
30.50	58.82	71.43	28.57	41.18	65.13	0.303
31.50	76.47	49.21	50.79	23.53	62.84	0.257
32.50	88.24	36.51	63.49	11.76	62.37	0.247
33.50	100.00	23.81	76.19	0.00	61.90	0.238

Youden's J coefficient

## DISCUSSION

The investigation of speech perception in schoolchildren with SLD and concomitant speech disorders assessed different auditory and perceptive-linguistic skills. This assessment was based on the simple speech processing model^(21)^ that assumes the existence of different levels of linguistic input and output processing. Thus, considering that the main linguistic input route is auditory, the model defined the following tasks for assessing input skills: auditory discrimination and auditory perception of speech sounds, identification of word structures, discrimination of actual words, receptive vocabulary, and phonological awareness^(22)^. Knowledge about these skills could explain how they are organized, temporally or hierarchically, and thus indicate where the link of acoustic or phonological information has been broken, impairing the full development of the speech sound system, the phonological information representations and, consequently, the processing of this information that should serve as a basis for learning to read and write^(21)^.

In this sense, hearing was assessed by investigating auditory perceptive skills of localization lateralization and temporal order. RGI and RGII showed similar performances^(26-28)^, and the better performance of the CG at this level of evaluation (localization and temporal order) was already expected^(27,29)^.

Studies indicate a strong association between Central Auditory Processing Disorders and Speech Sound Disorders^(5,18,27)^ and Reading and Writing Disorders^(29)^. According to these studies, processing information received through hearing plays a vital role in speech and language development, and impairment of these auditory mechanisms can contribute to the emergence of problems in speech learning and reading and writing. The integration between acoustic and phonological information may not have been broken at this speech perception level.

Data from the literature indicate that the ability to discriminate linguistic sounds and recognize phonemes consistently begins early despite a considerable variation in crucial acoustic parameters. Therefore, one would not expect to find school children over the age of 8 with difficulties in perceiving categories of linguistic sounds^(9-12,14-16)^. When categorically discriminating pseudowords with a Portuguese language structure in the TDP, the number of hits in the CG was higher only than that of RGII (p=0.035), and this did not differ from RGI. The worse performance of RGII in this test against the CG highlights the difficulty of categorical perception, also found in Brazilian schoolchildren.

Children who formed the RGII generally had more errors when discriminating phonemes in pseudowords in most of the test items. Analysis of the responses showed that most errors were found in pseudowords with complex syllables, such as consonant clusters. Analysis of these children’s speech shows that simplifying consonant clusters is the most frequent in this group (see supplementary material).

We could separate RGII from the other two groups when speech perception was analyzed using the TDP. The ROC curve indicated a cutoff value for the number of hits in the TDP. This value corresponded to 30.5 hits (out of 32 pairs of stimuli). Therefore, we estimated that accuracy values ​​lower than 30.5 in performance in the TDP would indicate the need to be aware of changes in speech production and perception. It would also indicate that it would be appropriate to include speech perception stimulation in therapeutic programs for SLD whenever identifying changes in speech sounds.

The integrity of the information archive and mental representations of phonemes and words is essential for objectifying words and achieving metalinguistic functions (such as phonological awareness) that underpin literacy and the correct learning and development of reading^(4,14)^. The perception of speech sounds is the beginning of this process.

However, speech disorders are not always evident when applying phonological assessment tests performed only with isolated words. Furthermore, it should be considered that school-age children, mainly because they are older, generally do not display severe speech sound disorders^(14)^. Information about previous speech development can also contribute to a better understanding of the condition^(16)^. Increasing the sample of children with more severe speech disorders could differentiate and classify each group evaluated more robustly.

## CONCLUSION

The ability to discriminate phonemes in pseudowords differentiated the RGII students, thus characterizing this group of children with Learning Disorders associated with Persistent Speech Sound Disorders. We could characterize this group of students with some hits lower than 30.5, considering the 34 items proposed in the task of categorical discrimination of phonemes in pseudowords. In other words, Persistent Speech Sound Disorder deteriorated the speech perception performance of children with Learning Disorders.

## Supplementary Material

Supplementary material accompanies this paper.

Chart S1 Evaluation Data of Students in the Control Group (CG).

Chart S2 Evaluation Data of Students in the SRD and SRD + PSSD Research Groups.

Table S1 Descriptive Summary of Age in the RGI, RGII, and CG Groups.

Table S2 Frequency and Percentage Distributions by Gender in the RGI, RGII, and CG Groups.

Table S3 Frequency and Percentage Distributions by School Year in the RGI, RGII, and CG Groups.

This material is available as part of the online article from https://doi.org/10.1590/2317-1782/20242024034en
