# Supplementary material for: Speech perception in the Specific Learning Disorder with and without Persistent Speech Sound Disorder
Source: Codas. 2024 Oct 4;36(5):e20240034. doi: 10.1590/2317-1782/20242024034en (PMC11534149; doi:10.1590/2317-1782/20242024034en)
Supplement: Chart S1 [file codas-36-5-e20240034-Suppl.pdf]

Chart S1 - Evaluation Data of Students in the Control Group (GC)

ABFW - Naming (%) - GC																	
N	Liq_ Simp	Cons_ ClS	Final_ CD	Plos_ Voic	Fric_ Voic	Plo_ Dev	Fric_ Dev	Syl_ Red	Cons_ Harm	Fri_ Stop	Vel_ Back	Pal_ Back	Vel_ Front	Pal_ Front	others	PCC	Classif.
1	0	0	0	0	0	0	0	0	0	0	0	0	0	0	0	100	No alt
2	0	0	0	0	0	0	0	0	0	0	0	0	0	0	0	100	No alt
3	0	0	0	0	0	0	0	0	0	0	0	0	0	0	0	100	No alt
4	0	0	0	0	0	0	0	0	0	0	0	0	0	0	0	100	No alt
5	0	0	0	0	0	0	0	0	0	0	0	0	0	0	0	100	No alt
6	0	0	0	0	0	0	0	0	0	0	0	0	0	0	0	100	No alt
7	0	0	0	0	0	0	0	0	0	0	0	0	0	0	0	100	No alt
8	0	0	0	0	0	0	0	0	0	0	0	0	0	0	0	100	No alt
9	0	0	0	0	0	0	0	0	0	0	0	0	0	0	0	100	No alt
10	0	0	0	0	0	0	0	0	0	0	0	0	0	0	0	100	No alt
11	0	0	0	0	0	0	0	0	0	0	0	0	0	0	0	100	No alt
12	0	0	0	0	0	0	0	0	0	0	0	0	0	0	0	100	No alt
13	0	0	0	0	0	0	0	0	0	0	0	0	0	0	0	100	No alt
14	0	0	0	0	0	0	0	0	0	0	0	0	0	0	0	100	No alt
15	0	0	0	0	0	0	0	0	0	0	0	0	0	0	0	100	No alt
16	0	0	0	0	0	0	0	0	0	0	0	0	0	0	0	100	No alt
17	0	0	0	0	0	0	0	0	0	0	0	0	0	0	0	100	No alt
18	0	0	0	0	0	0	0	0	0	0	0	0	0	0	0	100	No alt
19	0	0	0	0	0	0	0	0	0	0	0	0	0	0	0	100	No alt
20	0	0	0	0	0	0	0	0	0	0	0	0	0	0	0	100	No alt
21	0	0	0	0	0	0	0	0	0	0	0	0	0	0	0	100	No alt
22	0	0	0	0	0	0	0	0	0	0	0	0	0	0	0	100	No alt
23	0	0	0	0	0	0	0	0	0	0	0	0	0	0	0	100	No alt
24	0	0	0	0	0	0	0	0	0	0	0	0	0	0	0	100	No alt
25	0	0	0	0	0	0	0	0	0	0	0	0	0	0	0	100	No alt
26	0	0	0	0	0	0	0	0	0	0	0	0	0	0	0	100	No alt
27	0	0	0	0	0	0	0	0	0	0	0	0	0	0	0	100	No alt
28	0	0	0	0	0	0	0	0	0	0	0	0	0	0	0	100	No alt
29	0	0	0	0	0	0	0	0	0	0	0	0	0	0	0	100	No alt
30	0	0	0	0	0	0	0	0	0	0	0	0	0	0	0	100	No alt
31	0	0	0	0	0	0	0	0	0	0	0	0	0	0	0	100	No alt
32	0	0	0	0	0	0	0	0	0	0	0	0	0	0	0	100	No alt
33	0	0	0	0	0	0	0	0	0	0	0	0	0	0	0	100	No alt
34	0	0	0	0	0	0	0	0	0	0	0	0	0	0	0	100	No alt
35	0	0	0	0	0	0	0	0	0	0	0	0	0	0	0	100	No alt
36	0	0	0	0	0	0	0	0	0	0	0	0	0	0	0	100	No alt
37	0	0	0	0	0	0	0	0	0	0	0	0	0	0	0	100	No alt
38	0	0	0	0	0	0	0	0	0	0	0	0	0	0	0	100	No alt
39	0	0	0	0	0	0	0	0	0	0	0	0	0	0	0	100	No alt

40	0	0	0	0	0	0	0	0	0	0	0	0	0	0	0	100	No alt
41	0	0	0	0	0	0	0	0	0	0	0	0	0	0	0	100	No alt
42	0	0	0	0	0	0	0	0	0	0	0	0	0	0	0	100	No alt
43	0	0	0	0	0	0	0	0	0	0	0	0	0	0	0	100	No alt
44	0	0	0	0	0	0	0	0	0	0	0	0	0	0	0	100	No alt
45	0	0	0	0	0	0	0	0	0	0	0	0	0	0	0	100	No alt
46	0	0	0	0	0	0	0	0	0	0	0	0	0	0	0	100	No alt
47	0	0	0	0	0	0	0	0	0	0	0	0	0	0	0	100	No alt
48	0	0	0	0	0	0	0	0	0	0	0	0	0	0	0	100	No alt

Caption: Liq_Simp = Liquid Simplification; Cons_ClS = Consonant Cluster Simplification; Final_CD = Final Consonant Deletion; Plos_Voic= Plosive Voicing; Fric_Voic= Fricative Voicing; Plo_Dev = Plosive Devoicing; Fric_Dev = Fricative Devoicing; Syl_Red = Syllable Reduction; Cons_Harm = Consonant Harmony; Fric_Stop = Fricative Stopping; Vel = Velar Backing; Pal_Back = Palatal Backing; Vel_Front = Velar Fronting; Pal_Front = Palatal Fronting; PCC = Percentage of Correct Consonants; Classif = Classification; No alt = No alteration

Chart S2. Evaluation Data of Students in the TA and TA+TSFP Research Groups

ABFW - Naming (%) – GPI e GPII																	
N	Liq_Simp	Cons_ClS	Final_CD	Plos_Voic	Fric_Voic	Plos_Dev	Fric_Dev	Syl_Red	Cons_Harm	Fric_Stop	Vel_Back	Pal_Back	Vel_Front	Pal_Front	outros	PCC	Classif.
1	0	0	0	0	0	0	0	0	0	0	0	0	0	0	0	100	No alt
2	0	0	0	0	0	0	0	0	0	0	0	0	0	0	0	100	No alt
3	0	0	0	0	0	0	0	0	0	0	0	0	0	0	0	100	No alt
4	0	0	0	0	0	0	0	0	0	0	0	0	0	0	0	100	No alt
5	0	0	0	0	0	0	0	0	0	0	0	0	0	0	0	100	No alt
6	0	0	0	0	0	0	0	0	0	0	0	0	0	0	0	100	No alt
7	0	0	0	0	0	0	0	0	0	0	0	0	0	0	0	100	No alt
8	0	0	0	0	0	0	0	0	0	0	0	0	0	0	0	100	No alt
9	0	0	0	0	0	0	0	0	0	0	0	0	0	0	0	100	No alt
10	0	0	0	0	0	0	0	0	0	0	0	0	0	0	0	100	No alt
11	0	0	0	0	0	0	0	0	0	0	0	0	0	0	0	100	No alt
12	0	0	0	0	0	0	0	0	0	0	0	0	0	0	0	100	No alt
13	0	0	0	0	0	0	0	0	0	0	0	0	0	0	0	100	No alt
14	0	0	0	0	0	0	0	0	0	0	0	0	0	0	0	100	No alt
15	0	0	0	0	0	0	0	0	0	0	0	0	0	0	0	100	No alt
16	0	12,5	0	0	0	0	0	0	0	0	0	0	0	0	0	98,9	Mild
17	100	100	20	0	0	0	0	2,9	0	0	0	0	0	60	0	72,2	Mild. Mod.
18	0	0	20	0	0	0	0	2,9	0	0	0	0	0	0	1	96,6	Mild
19	0	12,5	0	0	0	0	0	0	0	0	0	0	0	0	0	98,9	Mild
20	0	12,5	0	0	0	0	0	0	0	0	0	0	0	0	0	98,9	Mild
21	0	12,5	0	0	0	0	0	0	0	0	0	0	0	0	0	98,9	Mild
22	0	0	40	0	0	0	0	0	0	0	0	0	0	0	0	97,7	Mild
23	0	0	40	0	0	0	0	0	0	0	0	0	0	0	0	97,7	Mild
24	0	12,5	0	0	0	0	0	0	0	0	0	0	0	0	0	98,9	Mild
25	0	12,5	0	0	0	0	0	0	0	0	0	0	0	0	0	98,9	Mild
26	0	12,5	0	0	0	0	0	0	0	0	0	0	0	0	0	98,9	Mild
27	0	12,5	0	0	0	0	0	0	0	0	0	0	0	0	0	98,9	Mild
28	100	100	20	0	0	0	0	2,9	0	0	0	0	0	60	0	72,2	Mild Mod.
29	0	12,5	0	0	0	0	0	0	0	0	0	0	0	0	0	98,9	Mild
30	0	12,5	0	0	0	0	0	0	0	0	0	0	0	0	0	98,9	Mild
31	100	100	20	0	0	0	0	2,9	0	0	0	0	0	60	0	72,2	Mild Mod.
32	0	12,5	0	0	0	0	0	0	0	0	0	0	0	0	0	98,9	Mild

Caption: Liq_Simp = Liquid Simplification; Cons_ClS = Consonant Cluster Simplification; Final_CD = Final Consonant Deletion; Plos_Voic= Plosive Voicing; Fric_Voic= Fricative Voicing; Plo_Dev = Plosive Devoicing; Fric_Dev = Fricative Devoicing; Syl_Red = Syllable Reduction; Cons_Harm = Consonant Harmony; Fric_Stop = Fricative Stopping; Vel = Velarization; Pal_Back = Palatal Backing; Vel_Front = Velar Fronting; Pal_Front = Palatal Fronting; PCC = Percentage of Correct Consonants; Classif = Classification; No alt = No alteration

Table S1 - Descriptive Summary of Age in the GPI, GPII, and GC Groups

Group	N	Mean	Standard Deviation	Minimum	Median	Maximum
GPI	15	9,5	1,4	8	9	11
GPII	17	9,4	1,0	8	9	11
GC	48	9,2	0,7	8	9	10
Total	80	9,3	0,9	8	9	11

Kruskal-Wallis Test; significance level = p<0,05.

Table S2 - Frequency and Percentage Distributions by Gender in the GPI, GPII, and GC Groups

Group	Sex		Total
	F	M	
GPI	8	7	15
	53,3%	46,7%	100,0%
GPII	5	12	17
	29,4%	70,6%	100,0%
GC	27	21	48
	56.3%	43.8%	100%
Total	40	40	80
	50%	50%	100%

Chi-Square Test; significance level = p<0,05.

Table S3 - Frequency and Percentage Distributions by School Year in the GPI, GPII, and GC Groups

Group	School grade					Total
	2º	3º	4º	5º	6º	
GPI	0	6	2	2	5	15
	0,0%	40,0%	13,3%	13,3%	33,3%	100,0%
GPII	1	8	4	2	2	17
	5,9%	47,1%	23,5%	11,8%	11,8%	100,0%
GC	0	14	23	11	0	48
	0,0%	29,2%	47,9%	22,9%	0,0%	100,0%
Total	1	28	29	15	7	80
	1,3%	35,0%	36,3%	18,8%	8,8%	100,0%

Fisher and van Belle's Likelihood Ratio Test; significance level = p<0,05.

Quadro 1 - Dados da avaliação dos escolares que compuseram o grupo controle (GC)

**ABFW - Nomeação (%) - GC**																	
N	Simp_Liq	Simp_Ec	Simp_Cf	Son_Plos	Son_Fric	Ensur_Plo	Ensur_Fri	Red_síl	Harm_Cons	Plos_Fric	Post_Vel	Post_Pal	Front_Vel	Front_Pal	outros	PCC	Classif.
1	0	0	0	0	0	0	0	0	0	0	0	0	0	0	0	100	Sem alt
2	0	0	0	0	0	0	0	0	0	0	0	0	0	0	0	100	Sem alt
3	0	0	0	0	0	0	0	0	0	0	0	0	0	0	0	100	Sem alt
4	0	0	0	0	0	0	0	0	0	0	0	0	0	0	0	100	Sem alt
5	0	0	0	0	0	0	0	0	0	0	0	0	0	0	0	100	Sem alt
6	0	0	0	0	0	0	0	0	0	0	0	0	0	0	0	100	Sem alt
7	0	0	0	0	0	0	0	0	0	0	0	0	0	0	0	100	Sem alt
8	0	0	0	0	0	0	0	0	0	0	0	0	0	0	0	100	Sem alt
9	0	0	0	0	0	0	0	0	0	0	0	0	0	0	0	100	Sem alt
10	0	0	0	0	0	0	0	0	0	0	0	0	0	0	0	100	Sem alt
11	0	0	0	0	0	0	0	0	0	0	0	0	0	0	0	100	Sem alt
12	0	0	0	0	0	0	0	0	0	0	0	0	0	0	0	100	Sem alt
13	0	0	0	0	0	0	0	0	0	0	0	0	0	0	0	100	Sem alt
14	0	0	0	0	0	0	0	0	0	0	0	0	0	0	0	100	Sem alt
15	0	0	0	0	0	0	0	0	0	0	0	0	0	0	0	100	Sem alt
16	0	0	0	0	0	0	0	0	0	0	0	0	0	0	0	100	Sem alt
17	0	0	0	0	0	0	0	0	0	0	0	0	0	0	0	100	Sem alt
18	0	0	0	0	0	0	0	0	0	0	0	0	0	0	0	100	Sem alt
19	0	0	0	0	0	0	0	0	0	0	0	0	0	0	0	100	Sem alt
20	0	0	0	0	0	0	0	0	0	0	0	0	0	0	0	100	Sem alt
21	0	0	0	0	0	0	0	0	0	0	0	0	0	0	0	100	Sem alt
22	0	0	0	0	0	0	0	0	0	0	0	0	0	0	0	100	Sem alt
23	0	0	0	0	0	0	0	0	0	0	0	0	0	0	0	100	Sem alt
24	0	0	0	0	0	0	0	0	0	0	0	0	0	0	0	100	Sem alt
25	0	0	0	0	0	0	0	0	0	0	0	0	0	0	0	100	Sem alt
26	0	0	0	0	0	0	0	0	0	0	0	0	0	0	0	100	Sem alt
27	0	0	0	0	0	0	0	0	0	0	0	0	0	0	0	100	Sem alt
28	0	0	0	0	0	0	0	0	0	0	0	0	0	0	0	100	Sem alt
29	0	0	0	0	0	0	0	0	0	0	0	0	0	0	0	100	Sem alt
30	0	0	0	0	0	0	0	0	0	0	0	0	0	0	0	100	Sem alt
31	0	0	0	0	0	0	0	0	0	0	0	0	0	0	0	100	Sem alt
32	0	0	0	0	0	0	0	0	0	0	0	0	0	0	0	100	Sem alt
33	0	0	0	0	0	0	0	0	0	0	0	0	0	0	0	100	Sem alt
34	0	0	0	0	0	0	0	0	0	0	0	0	0	0	0	100	Sem alt
35	0	0	0	0	0	0	0	0	0	0	0	0	0	0	0	100	Sem alt
36	0	0	0	0	0	0	0	0	0	0	0	0	0	0	0	100	Sem alt

37	0	0	0	0	0	0	0	0	0	0	0	0	0	0	0	100	Sem alt
38	0	0	0	0	0	0	0	0	0	0	0	0	0	0	0	100	Sem alt
39	0	0	0	0	0	0	0	0	0	0	0	0	0	0	0	100	Sem alt
40	0	0	0	0	0	0	0	0	0	0	0	0	0	0	0	100	Sem alt
41	0	0	0	0	0	0	0	0	0	0	0	0	0	0	0	100	Sem alt
42	0	0	0	0	0	0	0	0	0	0	0	0	0	0	0	100	Sem alt
43	0	0	0	0	0	0	0	0	0	0	0	0	0	0	0	100	Sem alt
44	0	0	0	0	0	0	0	0	0	0	0	0	0	0	0	100	Sem alt
45	0	0	0	0	0	0	0	0	0	0	0	0	0	0	0	100	Sem alt
46	0	0	0	0	0	0	0	0	0	0	0	0	0	0	0	100	Sem alt
47	0	0	0	0	0	0	0	0	0	0	0	0	0	0	0	100	Sem alt
48	0	0	0	0	0	0	0	0	0	0	0	0	0	0	0	100	Sem alt

Quadro 2. Dados da avaliação dos escolares que compuseram os grupos pesquisa TA e TA+TSFP

ABFW - Nomeação (%) – GPI e GPII																	
N	Simp_Liq	Simp_Ec	Simp_Cf	Son_Plos	Son_Fric	Ensur_Plo	Ensur_Fri	Red_síl	Harm_Cons	Plos_Fric	Post_Vel	Post_Pal	Front_Vel	Front_Pal	outros	PCC	Classif.
1	0	0	0	0	0	0	0	0	0	0	0	0	0	0	0	100	Sem.alt
2	0	0	0	0	0	0	0	0	0	0	0	0	0	0	0	100	Sem.alt
3	0	0	0	0	0	0	0	0	0	0	0	0	0	0	0	100	Sem.alt
4	0	0	0	0	0	0	0	0	0	0	0	0	0	0	0	100	Sem.alt
5	0	0	0	0	0	0	0	0	0	0	0	0	0	0	0	100	Sem.alt
6	0	0	0	0	0	0	0	0	0	0	0	0	0	0	0	100	Sem.alt
7	0	0	0	0	0	0	0	0	0	0	0	0	0	0	0	100	Sem.alt
8	0	0	0	0	0	0	0	0	0	0	0	0	0	0	0	100	Sem.alt
9	0	0	0	0	0	0	0	0	0	0	0	0	0	0	0	100	Sem.alt
10	0	0	0	0	0	0	0	0	0	0	0	0	0	0	0	100	Sem.alt
11	0	0	0	0	0	0	0	0	0	0	0	0	0	0	0	100	Sem.alt
12	0	0	0	0	0	0	0	0	0	0	0	0	0	0	0	100	Sem.alt
13	0	0	0	0	0	0	0	0	0	0	0	0	0	0	0	100	Sem.alt
14	0	0	0	0	0	0	0	0	0	0	0	0	0	0	0	100	Sem.alt
15	0	0	0	0	0	0	0	0	0	0	0	0	0	0	0	100	Sem.alt
16	0	12,5	0	0	0	0	0	0	0	0	0	0	0	0	0	98,9	Leve
17	100	100	20	0	0	0	0	2,9	0	0	0	0	0	60	0	72,2	lev. Mod.
18	0	0	20	0	0	0	0	2,9	0	0	0	0	0	0	1	96,6	leve
19	0	12,5	0	0	0	0	0	0	0	0	0	0	0	0	0	98,9	Leve
20	0	12,5	0	0	0	0	0	0	0	0	0	0	0	0	0	98,9	Leve
21	0	12,5	0	0	0	0	0	0	0	0	0	0	0	0	0	98,9	Leve
22	0	0	40	0	0	0	0	0	0	0	0	0	0	0	0	97,7	Leve
23	0	0	40	0	0	0	0	0	0	0	0	0	0	0	0	97,7	Leve
24	0	12,5	0	0	0	0	0	0	0	0	0	0	0	0	0	98,9	Leve
25	0	12,5	0	0	0	0	0	0	0	0	0	0	0	0	0	98,9	Leve
26	0	12,5	0	0	0	0	0	0	0	0	0	0	0	0	0	98,9	Leve
27	0	12,5	0	0	0	0	0	0	0	0	0	0	0	0	0	98,9	Leve
28	100	100	20	0	0	0	0	2,9	0	0	0	0	0	60	0	72,2	lev. Mod.
29	0	12,5	0	0	0	0	0	0	0	0	0	0	0	0	0	98,9	Leve
30	0	12,5	0	0	0	0	0	0	0	0	0	0	0	0	0	98,9	Leve
31	100	100	20	0	0	0	0	2,9	0	0	0	0	0	60	0	72,2	lev. Mod.
32	0	12,5	0	0	0	0	0	0	0	0	0	0	0	0	0	98,9	Leve

Tabela 1- Resumo descritivo da idade nos GPI, GPII e GC.

Grupo	N	Média	Desvio Padrão	Mínimo	Mediana	Máximo
GPI	15	9,5	1,4	8	9	11
GPII	17	9,4	1,0	8	9	11
GC	48	9,2	0,7	8	9	10
Total	80	9,3	0,9	8	9	11

Teste de Kruskal-Wallis; nível de significância = $p<0,05$.

Tabela 2- Distribuições de frequências e porcentagens segundo o sexo nos GPI, GPII e GC.

Grupo	Sexo		Total
	F	M	
GPI	8	7	15
	53,3%	46,7%	100,0%
GPII	5	12	17
	29,4%	70,6%	100,0%
GC	27	21	48
	56.3%	43.8%	100%
Total	40	40	80
	50%	50%	100%

Teste Quiquadrado; nível de significância = p<0,05.

Tabela 3- Distribuições de frequências e porcentagens por ano escolar em GPI, GPII e GC.

Grupo	Ano Escolar					Total
	2º	3º	4º	5º	6º	
GPI	0	6	2	2	5	15
	0,0%	40,0%	13,3%	13,3%	33,3%	100,0%
GPII	1	8	4	2	2	17
	5,9%	47,1%	23,5%	11,8%	11,8%	100,0%
GC	0	14	23	11	0	48
	0,0%	29,2%	47,9%	22,9%	0,0%	100,0%
Total	1	28	29	15	7	80
	1,3%	35,0%	36,3%	18,8%	8,8%	100,0%

Teste da Razão de Verossimilhanças de Fisher e van Belle; nível de significância = p<0,05.
