# Supplementary material for: Speech perception in the Specific Learning Disorder with and without Persistent Speech Sound Disorder
Source: Codas. 2024 Oct 4;36(5):e20240034. doi: 10.1590/2317-1782/20242024034en (PMC11534149; doi:10.1590/2317-1782/20242024034en)
Supplement: Translation

# Translation: Percepção de fala no transtorno específico da aprendizagem com e sem transtorno persistente dos sons da fala

**DOI:** 10.1590/2317-1782/20242024034pt

**Authors:** Mariana Martins Appezzato, Clara Regina Brandão de Avila

**Affiliations:** 1 Programa de Pós-graduação em Distúrbios da Comunicação Humana, Escola Paulista de Medicina – EPM, Universidade Federal de São Paulo – UNIFESP - São Paulo (SP), Brasil.; 2 Departamento de Fonoaudiologia, Escola Paulista de Medicina – EPM, Universidade Federal de São Paulo – UNIFESP - São Paulo (SP), Brasil.

**Keywords:** Percepção da Fala, Distúrbios da Fala, Transtorno de Aprendizagem Específico, Ensino Fundamental e Médio, Curva ROC

## Abstract

**Objetivo:**

Investigar se a habilidade de percepção de fala pode diferenciar escolares com Transtorno específico de aprendizagem (TAp) com e sem Transtorno Persistente dos Sons da Fala (TPSF).

**Método:**

Participaram da pesquisa 80 crianças, regularmente matriculadas no 2º (N=1), 3º (N=28), 4º (N=29), 5º (N=15), e 6º (N=7) anos, assim reunidos: Grupo Controle (N= 48) - sem queixas, sem alteração de fala; e Grupos Pesquisa (N = 32) - com TAp, sendo GPI (N = 15) sem TPSF e, GPII (N = 17), com TPSF. Duas provas avaliaram input auditivo: Avaliação simplificada do processamento auditivo; e Tarefa de percepção de pseudopalavras com estrutura da língua portuguesa - TDP. Os dados foram analisados pelos testes: Razão de Verossimilhanças, Kruskal-Wallis, Dunn com correção de Bonferroni, Mann-Whitney, correlação de Spearman, além da construção de uma curva ROC para obter um valor de corte para o número de acertos na prova de percepção de não palavras.

**Resultados:**

Controle e GPI mostraram maiores escores de acerto que GPII. Não houve diferença entre as distribuições de acertos do Controle e GPI e do GPI e GPII na TDP e o número de acertos do GC foi maior que o do GPII.

**Conclusão:**

A habilidade de discriminação de pseudopalavras diferenciou os escolares com TAp e TPSF das crianças sem TPSF, caracterizando, assim esse grupo por apresentar número de acertos menor que 30,5, considerada a tarefa proposta para discriminar pseudopalavras. Esses resultados sugerem que a presença do TPSF piorou o desempenho em percepção de fala dos escolares com TAp.

## INTRODUÇÃO

A capacidade de perceber um único fonema na fala encadeada é competência metalinguística que será demandada no aprendizado do princípio alfabético^(1,2)^ Alterações na produção, tanto quanto na percepção da fala, podem interferir na aquisição de contrastes fonológicos e na categorização das representações mentais de segmentos linguísticos, dificultando o desenvolvimento da consciência fonológica^(3-5)^. Evidências científicas indicam que é preciso considerar questões subjacentes a essa capacidade de manipular mentalmente, os sons da cadeia de fala^(6)^, sobretudo quando se avalia uma criança com queixa e sinais de algum Transtorno específico de aprendizagem – TAp^(7)^. Nesses casos, mais que pensar no déficit da capacidade metacognitiva de manipular fonemas e conectá-los às letras é preciso considerar as condições e características do arquivo de representações fonológicas advindo do desenvolvimento da fala^(8)^ e de como esta é organizada e percebida.

Por isso, não é incomum encontrar crianças que apresentam transtorno de leitura e/ou de escrita e manifestam, em coocorrência, ou, em associação, Transtorno dos sons da fala^(9-12)^. A base atual de evidências sugere que a presença de alterações dos sons da fala aumenta a propensão para empreender maiores esforços na aquisição de habilidades próprias da alfabetização^(13-15)^.

A presença de alteração da fala na época da alfabetização, evidencia sua persistência com possibilidade de interferir ou restringir a comunicação e com impacto negativo na interação social e/ou no sucesso acadêmico^(7,9)^.

O TAp pode exibir um espectro de déficits de habilidades cognitivo-linguísticas que se manifestam nos diferentes domínios da linguagem^(7)^. Não raramente, observam-se desde transtornos mais graves dos sons da fala^(14)^, até déficits subclínicos que envolvem erros específicos^(15)^, ou processos fonológicos ainda não solucionados^(9,16)^ acompanhando os TAp.

Uma vez que ainda são observados em idade escolar (tardiamente, em fase ulterior à de finalização das aquisições de fala) e, consideradas as características de sua produção, essas alterações de fala são consideradas Transtornos Persistentes dos Sons da Fala - TPFS^(15)^. Eles são o desfecho de um desenvolvimento que ocorreu diferentemente do esperado, tanto em tempo quanto em ordem de aquisições, ou ainda, com distorções^(15,16)^. Pesquisadores^(17)^ concordam que o risco de apresentar TAp aumenta conforme mais habilidades da linguagem e fala estejam alteradas em idades que precedem ou incidem à entrada no Ensino Fundamental. O TAp pode estar frequentemente associado a déficits do processamento auditivo e da percepção da fala^(18)^.

Os Transtornos específicos da aprendizagem, com prejuízo na leitura e na escrita^(7)^ têm como denominador comum déficits do processamento da informação fonológica que se manifestam em uma variedade de habilidades e tarefas para ler e escrever. Os mecanismos subjacentes que impulsionam esse déficit ainda não são totalmente conhecidos^(6)^. Estudos sobre a percepção de fala, nesses casos, são os menos frequentes. Então, parece importante conhecer qual mecanismo envolvido nos inputs sensoriais ou perceptivos auditivos é mais frágil na percepção da fala^(19)^, sobretudo quando alterações no sistema de fala estão presentes para além da idade esperada.

Alterações relacionadas às dificuldades de percepção de fala podem abarcar prejuízos: da discriminação sonora, ou seja, de atenção às diferenças acústicas dos sons da fala; da percepção categórica, ou classificação dos sons da fala em categorias de julgamento; ou da detecção de erros; ou da capacidade de perceber se um som de fala pertence, ou não, à sua língua; ou, ainda, de produzir palavras com base no idioma nativo^(19-21)^.

Déficits na percepção da fala são comumente relatados no TAp e a sobreposição de alterações de fala às de leitura devem piorar as condições de inputs auditivos, sobretudo para perceber as características acústicas dos fonemas^(6)^ e proceder a eventuais correções. Entretanto, as evidências longitudinais de que a pobre percepção de fala compromete a aprendizagem da leitura ainda são escassas.

Por esse motivo, este estudo tem como foco investigar certas características de percepção de fala de estudantes do Ensino Fundamental I, com diagnóstico de TAp. Alguns desses estudantes apresentaram à época da coleta, em associação ao quadro principal, TPSF e formaram um subgrupo de investigação.

Conduzida segundo um modelo psicolinguístico^(21)^ esta pesquisa avaliou a capacidade de percepção categórica dos sons da fala com a aplicação de uma lista de pseudopalavras para discriminação e classificação fonêmica^(22)^.

Examinou-se, então, a percepção de fala em diferentes perfis de déficit fonológico, em relação ao desenvolvimento normal. Assim, questionar se o TPSF pode piorar o desempenho em percepção de fala de estudantes com TAp definiu esta pesquisa. A partir dessa questão e das evidências da literatura levantou-se, então, a hipótese de que no TAp a capacidade de perceber categoricamente os sons da fala seja deficitária em relação a estudantes de desenvolvimento típico. E será pior quando em associação com o TSFP.

## **●** MÉTODO

Este foi um estudo de corte transversal e análise quantitativa, submetido (CAAE: 47313115.5.0000.5505) e aprovado (nº 1415919) pelo CEP-UNIFESP. Todos os participantes assinaram os Termos de Consentimento Livre e Esclarecido (TCLE), de Assentimento da Criança (TAC) e de Anuência da Escola (TAE).

### Participantes

A amostra de escolares foi constituída por conveniência e composta por 80 crianças (de 08 anos e 2 meses a 11 anos e 8 meses / Média = 9,3 anos; DP = 0,9) estudantes da rede pública de ensino do município de São Paulo. Estabeleceu-se como critério geral de inclusão na amostra, a matrícula regular do 2º ao 6º ano do Ensino Fundamental I de escola da rede pública e como critérios de exclusão na amostra, queixas ou indicadores de déficits sensoriais (auditivos ou visuais), de distúrbios neurológicos, de déficits cognitivos, mencionadas pelos professores ou por seus pais; anomalias ou alterações crânio-maxilo-faciais; ausência de Reflexo Cócleo-Palpebral na Avaliação Simplificada do Processamento Auditivo^(23)^; e ausência do preenchimento do TCLE ou do TAC assinado.

As 80 crianças escolares foram agrupadas da seguinte forma: Grupo de Controle - GC (n=48 escolares com desenvolvimento típico de aprendizagem) e Grupo de Pesquisa - GP (n=32 escolares com Transtorno específico de aprendizagem). O GP foi reagrupado em: GPI (n=15 escolares com TAp) e GPII (n= 17 escolares com TAp e TPSF). Para estudar a característica da percepção auditivo-linguística na presença do TPSF o desempenho desse grupo foi comparado com o GPI (TAp) e com o GC.

Os participantes dos GP estavam, à época da coleta, em atendimento no Ambulatório de Avaliação e Terapia dos Transtornos de Leitura e Escrita e receberam diagnóstico multidisciplinar de TAp. O GPII sobrepunha em coocorrência o diagnóstico fonoaudiológico de TPSF.

### Procedimentos

As seguintes provas e testes foram aplicados na avaliação de todos os participantes:

- Avaliação dos Sons da Fala ABFW - Fonologia^(24)^ - foram aplicadas as provas de nomeação e imitação para levantamento do inventário fonético, investigação da ocorrência dos processos fonológicos e o cálculo do índice de Porcentagem de Consoantes Corretas^(25)^. A aplicação permitiu identificar, classificar e separar, entre as crianças do GP, aquelas que apresentavam alterações dos sons da fala em concomitância com o TAp. (Dados descritivos em material suplementar – Quadros S1 e S2);
- para avaliar o primeiro nível de input auditivo-perceptivo, após o sensorial^(7)^, aplicou-se a Avaliação Simplificada do Processamento Auditivo - ASPAC^(23)^, uma vez que esta avalia, também, a percepção de sons linguísticos. A investigação do RCP permitiu a exclusão de crianças com risco de apresentarem problemas auditivos que poderiam prejudicar os resultados da aplicação das provas seguintes.
- Tarefa de Percepção de Pseudopalavras com Estrutura da Língua Portuguesa - TDP^(22)^: sua aplicação permitiu avaliar a capacidade de discriminação de sons da fala (sem referência à representação lexical) e a habilidade de percepção categórica dos sons da fala^(4,20)^. Dezoito itens de teste compuseram essa tarefa^(22)^.

Esses dois procedimentos de avaliação da percepção auditivo-linguística foram aplicados e analisados, respectivamente, segundo os procedimentos e parâmetros descritos pelas autoras^(22,23)^.

A coleta de dados foi realizada individualmente, em uma única sessão de avaliação que durou 50 minutos. As crianças do GC foram avaliadas na sala de recursos da escola, disponibilizada pela direção. O GP foi avaliado em sala silenciosa do Ambulatório.

O Teste de Kruskal-Wallis (p<0,05) e o teste do Quiquadrado (p<0,05) analisaram, respectivamente, as distribuições da idade e de probabilidade do sexo, nos três grupos. As medidas de tendência central e de dispersão de acertos na Tarefa de Percepção de Pseudopalavras com Estrutura da Língua Portuguesa - TDP de acordo com o grupo, bem como a comparação dos grupos foram estudadas por meio da ANOVA com um fator independente ou teste de Kruskal Wallis. Para caracterizar os Grupos, procedeu-se à investigação da sensibilidade e especificidade para a identificação de indivíduos com TAp com TPSF e estabelecimento do ponto de corte de maior equilíbrio entre estes dois parâmetros. Para o cálculo da curva ROC utilizou-se o total de acertos na TDP para identificar indivíduos do GPII. GC e GPI foram reunidos em um único grupo, pois não apresentaram diferença nesta prova. O valor de significância estatística adotado na análise dos dados foi igual a 5% (p ≤ 0,05). Utilizou-se o software SPSS Statistics, versão 25.0 (IBM Corp., Armonk, NY, EUA). Para o cálculo dos intervalos de confiança de 95% foi utilizado o método de viés corrigido e acelerado com base em 2000 amostras *Bootstrap*. A análise foi realizada com o auxílio dos aplicativos SPSS – versão 18, Minitab – versão 18 e R 3.5.1.

## RESULTADOS

Os três grupos eram semelhantes (p = 0,700) quanto a distribuições da idade. As crianças do GC estiveram distribuídas do 3º ao 5º ano; as do GPI do 3º ao 6º, e as do GPII do 2º ao 6º ano. Por outro lado, as distribuições das porcentagens de crianças, por ano escolar, nos três grupos, foram diferentes (conforme material suplementar – Tabelas S1, S2 e S3).

A comparação do desempenho em percepção auditiva, nas tarefas de Localização Sonora e Memória de Sequência de Sons Não Linguísticos e Sons Linguísticos (Tabela 1) não mostrou diferença entre os Grupos Pesquisa. Para análises posteriores, ambos os grupos pesquisa foram considerados um único que foi comparado ao GC. Este mostrou melhor desempenho nessas três tarefas de percepção auditiva.

**Tabela 1:** Distribuições de frequências e porcentagens do total de acertos nos Testes de Localização Sonora, de Memória de Sequência de Sons Não Linguísticos, de Memória de Sequência de Sons Linguísticos no GPI, GPII e GC

Provas		GPI	GPII	GC	Total
	Número de acertos				
LS	0	2	0	0	2
		13,30%	0,00%	0,00%	2,50%
	1	13	17	0	30
		86,70%	100%	0,00%	37,50%
	3	0	0	3	3
		0,00%	0,00%	6,30%	3,80%
	4	0	0	13	13
		0,00%	0,00%	27,10%	16,30%
	5	0	0	32	32
		0,00%	0,00%	66,70%	40,00%
TMSSNL	0	1	2	0	3
		6,70%	11,80%	0,00%	3,80%
	1	14	15	3	32
		93,30%	88,20%	6,30%	40,00%
	2	0	0	15	15
		0,00%	0,00%	31,30%	18,80%
	3	0	0	30	30
		0,00%	0,00%	62,50%	37,50%
TMSSL	0	2	2	0	4
		13,30%	11,80%	0,00%	5,00%
	1	13	15	0	28
		86,70%	88,20%	0,00%	35,00%
	2	0	0	13	13
		0,00%	0,00%	27,10%	16,30%
	3	0	0	35	35
		0,00%	0,00%	72,90%	43,80%
	Total	15	17	48	80
		100,00%	100%	100%	100,00%

Teste de Razão de Verossimilhança (p=0,424): GP1=GP2 | Teste Quiquadrado: GC>GP (p<0,001).

**Legenda:** LS = Localização Sonora; TMSSNL = Teste de Memória de Sequência de Sons Não Linguísticos; TMSSL = Teste de Memória de Sequência de Sons Linguísticos

Os Grupos GC e GP mostraram desempenho diferente na TDP (Tabela 2). O cálculo do tamanho do efeito da diferença entre os grupos por meio do coeficiente r indicou que essa diferença, de fato, foi encontrada entre os grupos GC e GPII (p = 0,035, r = 0,313).

**Tabela 2:** Valores descritivos e análise comparativa dos grupos em relação ao número de acertos na TDP

Prova	Grupo	n	Média	DP	Mediana	Mín.	Máx.	p
TDP	GC	48	31,00 [29,92, 31,94]	3,48	31,50 [31,00, 32,50]	16,00	34,00	**0,039** ^*^
	GPI	15	30,47 [27,80, 32,27]	4,47	31,00 [31,00, 33,00]	16,00	34,00	
	GPII	17	29,35 [28,12, 30,59]	2,76	30,00 [29,00, 30,00]	24,00	33,00	

(^a^ )Teste de Kruskal-Wallis

* Valor estatisticamente significante no nível de 5% (p ≤ 0,05).

**Legenda:** DP: Desvio padrão; Mín.: Mínimo; Máx.: Máximo

Uma vez que os grupos GC e GPI não apresentaram diferença na prova TDP, foram reunidos como um único grupo. A curva ROC caracterizou a capacidade do número total de acertos em TDP de identificar indivíduos do GPII.

Um valor de área sob a curva (AUC) de 0,698, indicou que um indivíduo do GP II (com TAp e TPSF) tem 69,8% de probabilidade de ter menor número de acertos na TDP, quando comparado a um escolar do GPI ou do GC, ou seja, sem Transtorno Persistente dos Sons da Fala (Figura 1).

**Figura 1:** Curva ROC para o número total de acertos na TDP. A linha tracejada indica um teste hipotético sem habilidade discriminante. AUC – 0,698 (IC 95%: [0,570, 0,827]).

O ponto de corte com maior equilíbrio entre sensibilidade e especificidade para o número de acertos na TDP foi 30,50 no qual a sensibilidade foi de 58,82% e a especificidade de 71,43%. (Tabela 3).

**Tabela 3:** Pontos de corte para o número de acertos na TDP e valores de sensibilidade, especificidade e eficiência associados

Ponto de corte	Sensibilidade (%)	Especificidade (%)	Falso positivo (%)	Falso negativo (%)	Eficiência (%)	J
18,50	0,00	96,83	3,17	100,00	48,41	-0,032
22,50	0,00	95,24	4,76	100,00	47,62	-0,048
24,50	5,88	93,65	6,35	94,12	49,77	-0,005
26,00	17,65	93,65	6,35	82,35	55,65	0,113
27,50	17,65	92,06	7,94	82,35	54,86	0,097
28,50	35,29	84,13	15,87	64,71	59,71	0,194
29,50	47,06	79,37	20,63	52,94	63,21	0,264
30,50	58,82	71,43	28,57	41,18	65,13	0,303
31,50	76,47	49,21	50,79	23,53	62,84	0,257
32,50	88,24	36,51	63,49	11,76	62,37	0,247
33,50	100,00	23,81	76,19	0,00	61,90	0,238

Coeficiente J de Youden

## **●** DISCUSSÃO

A investigação da condição de percepção de fala em crianças escolares com TAp e alterações de fala concomitantes avaliou diferentes habilidades auditivas e perceptivo-linguísticas. Essa avaliação baseou-se no modelo simples de processamento de fala^(21)^ que pressupõe a existência de diferentes níveis de processamento de input e output linguístico. Assim, considerando que a principal via de entrada linguística é a auditiva o modelo definiu, para a avaliação das habilidades de input, as tarefas: discriminação auditiva e percepção auditiva de sons da fala; identificação de estruturas de palavras; discriminação de palavras reais; vocabulário receptivo; consciência fonológica^(22)^. O conhecimento sobre essas habilidades poderia explicar como elas se organizam, temporal ou hierarquicamente e, assim, indicar onde o elo de informações acústicas e/ou fonológicas se rompeu, prejudicando o pleno desenvolvimento do sistema de sons da fala, das representações das informações fonológicas e, em consequência, o processamento dessas informações que devem servir de base para o aprendizado da leitura e da escrita^(21)^.

Nesse sentido avaliou-se a audição desde a investigação de habilidades perceptivas auditivas de localização e lateralização e ordenação temporal GPI e GPII mostraram desempenhos semelhantes^(26,27,28)^ e o melhor desempenho do GC nesse nível de avaliação (de localização e ordenação temporal) já era esperado^(27,29)^. Estudos indicam haver forte associação entre os Transtornos do Processamento Auditivo Central e os Transtornos dos sons da fala ^(5,18,27^ ) e os de Leitura e Escrita ^(29)^. Segundo esses estudos, processar as informações recebidas por meio da audição tem função importante no desenvolvimento de fala e de linguagem, e o prejuízo nesses mecanismos auditivos pode contribuir para o aparecimento de problemas do aprendizado da fala tanto quanto da leitura e da escrita. Possivelmente, não foi nesse nível de percepção de fala que a integração entre as informações acústicas e as fonológicas se rompeu.

Os dados da literatura alertam para o início precoce do desenvolvimento da habilidade de discriminar sons linguísticos e da capacidade de reconhecer fonemas de maneira consistente a despeito de uma grande variação em parâmetros acústicos cruciais. Portanto, não é esperado encontrar crianças escolares, com idades acima de 08 anos, com dificuldades para perceber categorias dos sons linguísticos^(9,10-16)^. Ao discriminarem categoricamente pseudopalavras com estrutura da língua portuguesa na TDP, o número de acertos no GC foi maior, apenas, que o do GPII (p=0,035) e este não se diferenciou do GPI. O pior desempenho do GPII nessa prova, em relação ao GC evidencia a dificuldade de percepção categórica, também presente em escolares brasileiros.

De forma geral, as crianças que compuseram o GPII apresentaram mais erros ao discriminar fonemas em pseudopalavras, em grande parte dos itens do teste. A análise das respostas mostrou que a maioria dos erros esteve presente em pseudopalavras com sílabas complexas, como o encontro consonantal. A análise da fala dessas crianças mostra a ocorrência do processo de simplificação de encontro consonantal como a mais frequente nesse Grupo (material suplementar – Quadro S2).

De qualquer forma foi possível separar GPII dos outros dois grupos, quando se analisou a percepção de fala por meio da TDP. A curva ROC indicou um valor de corte para o número de acertos na TDP. Esse valor correspondeu a 30,5 acertos (de 32 pares de estímulos). Por isso, estima-se que valores de acerto inferiores a 30,5, de desempenho na TDP indicariam a necessidade de estar atento à presença de alterações tanto de produção quanto de percepção da fala. Indicaria, também, que seria apropriado incluir a estimulação da percepção de fala em programas terapêuticos para TAp, sempre que alterações dos sons da fala estiverem presentes.

A integridade do arquivo de informações e representações mentais de fonemas e vocábulos é imperiosa para a objetivação das palavras e o alcance de funções metalinguísticas (como a consciência fonológica) que fundamentarão a alfabetização e o aprendizado e desenvolvimento corretos da leitura^(4,14)^. A percepção dos sons da fala é o início desse processo.

Contudo, nem sempre as alterações de fala são muito evidentes na aplicação de testes de avaliação fonológica realizados apenas com palavras isoladas. Além disso, deve-se considerar que as crianças na idade escolar, principalmente por serem mais velhas, em geral, não apresentam transtornos graves dos sons da fala^(14)^. Informações sobre o desenvolvimento pregresso da fala podem, igualmente, contribuir com o melhor entendimento do quadro^(16)^. O aumento da amostra de crianças que apresentem quadros mais graves de alterações da fala, talvez possa diferenciar e classificar de forma mais robusta cada grupo avaliado.

## CONCLUSÃO

A habilidade de discriminar fonemas em pseudopalavras diferenciou os estudantes do GPII, caracterizando, assim, esse grupo de crianças com Transtornos de Aprendizagem associado a Transtornos dos Sons da Fala Persistente. Foi possível caracterizar esse grupo de estudantes com número de acertos menor que 30,5, considerados os 34 itens propostos na tarefa de discriminação categórica de fonemas em pseudopalavras. Ou seja, a presença do Transtorno dos Sons da Fala Persistente piorou o desempenho em percepção da fala das crianças com Transtornos de Aprendizagem.

## Material Suplementar

Este artigo acompanha material suplementar.

Quadro S1 Dados da avaliação dos escolares que compuseram o grupo controle (GC)

Quadro S2 Dados da avaliação dos escolares que compuseram os grupos pesquisa TA e TA+TSFP

Tabela S1 Resumo descritivo da idade nos GPI, GPII e GC.

Tabela S2 Distribuições de frequências e porcentagens segundo o sexo nos GPI, GPII e GC.

Tabela S3 Distribuições de frequências e porcentagens por ano escolar em GPI, GPII e GC.

Este material está disponível como parte da versão online do artigo na página https://doi.org/10.1590/2317-1782/20242024034pt
